# Supplementary material for: Optimizing young tennis players’ development: Exploring the impact of emerging technologies on training effectiveness and technical skills acquisition
Source: PLoS One. 2024 Aug 7;19(8):e0307882. doi: 10.1371/journal.pone.0307882 (PMC11305591; doi:10.1371/journal.pone.0307882)
Supplement: Attachment — Submitted filename: peer-review_pone.docx [file pone.0307882.s002.docx]

**Review comment**

Thank you for submitting your manuscript titled "Optimizing Young Tennis Players' Development: Exploring the Impact of Emerging Technologies on Training Effectiveness and Technical Skills Acquisition" to our journal. Your research presents a comprehensive examination of how various emerging technologies can enhance the training and development of young tennis players. The focus on AI-powered coaching systems, virtual reality training environments, and wearable sensors provides valuable insights into modern training methodologies. However, several aspects of the manuscript may benefit from further elaboration and clarification.

I recommend several revisions and clarifications to enhance the manuscript's quality and readability:

Specific Comments:

**Abstract**：Could you expand on the results in the abstract to include specific statistical details? This would provide a clearer and more comprehensive picture of how each technology influenced technical skills acquisition and the extent to which personalized learning did not have a significant mediation effect.

**Introduction**：It is recommended to reorganize the introduction. Could expanding the discussion on existing research and literature more clearly demonstrate the specific knowledge gaps your study addresses? There is a need to more tightly connect the shortcomings of traditional methods with the advantages of new technologies. Please clearly indicate how each emerging technology overcomes specific limitations of traditional training, including detailed application examples, thereby enhancing the article's arguments.

1. "Physical conditioning is the other sporting attribute critical for tennis players. Many sports activities are physical, and players require proper strength and conditioning to ensure competition." What specific elements does 'physical conditioning' comprise? How does it impact the athletic performance of tennis players?
2. "Training through exercise and workouts among young players helps in developing speed, agility, strength, and coordination." Could you specify which types of 'exercise and workouts' are included here? Please provide supporting references.
3. "Traditional tennis sport training methods have long played a critical role in athlete development." Could you detail what encompasses traditional training methods? Please include necessary references.
4. "However, emerging issues in sports training activities depict challenges inherent in traditional training methods." Could you provide a detailed explanation of "emerging issues," offering specific examples or data that support these challenges in traditional training methods?
5. "Traditional training methods need help in replicating the real game scenarios, which can lead to differences in players' performance." You noted challenges with replicating real game scenarios using traditional training methods. Could you elaborate on how these limitations specifically affect the skill development of young tennis players?
6. "High physical activities during training increases the risk." Could you describe what types of injury risks are associated with high-intensity physical activities?
7. "Also, traditional training practices have limited rehabilitation integration." "Limited player rehabilitation integration can lead to slowed recovery from injury and return to top performance." How does traditional training impact rehabilitation integration? Is there any data or research to support this?
8. "Some essential technologies include an AI-powered coaching system, which utilizes artificial intelligence to help coaches analyze and track players' performance." Could you provide specific examples of how AI systems analyze and track performance, and how this data is subsequently utilized to develop training plans?
9. "Virtual Reality (VR) training environment is the other emerging technology in sports training that offers a dynamic and distraction-free environment." Can you provide examples of how VR technology creates a "distraction-free" environment and how this specifically benefits technical skill training?
10. "Wearable Sensor Technology in training offers players improved performance due to the ability to track health and fitness data, which is then used to enhance the individuals' athletic performance." How does Wearable Sensor Technology enhance athletic performance through tracking health and fitness data? Please provide a detailed explanation.

**Methods**

1. "From AI-powered coaching systems to virtual reality training environments and wearable sensors, training technologies play critical roles in skill refinement..."Could you provide more specific examples of how each technology—AI systems, VR environments, and wearable sensors—concretely contributes to skill refinement in tennis training? Are there particular skills these technologies target more effectively.
2. "The diffusion of Innovation Theory explains the phenomenon of emerging technologies in tennis training as an idea that gains traction and spreads to society." How does the Diffusion of Innovation Theory specifically apply to the adoption rates of these technologies among tennis coaches and athletes? Can you elaborate on how the characteristics of early adopters versus laggards manifest in this particular sports training context?
3. "Various studies opine that those technologies significantly influence tennis athletes' training activities..."Are there contrasting studies that may not support the effectiveness of these technologies in enhancing tennis training? Including a range of studies might provide a balanced view and strengthen the argument for the adoption of these technologies.
4. "The research investigated optimizing young tennis players' development... The study targeted a representative sample of a maximum of 400 young tennis players." How was the sample size of 400 young tennis players determined to be sufficient for this study? What statistical power analysis was conducted to ensure this sample size could adequately detect the expected effects?
5. "Primary data was collected from the respondents using a structured questionnaire... The questions on latent variables were framed on a Likert scale format." How were the questionnaire items developed and validated to ensure they accurately measure the constructs of interest, such as training effectiveness and technical skill acquisition? Were any pilot tests conducted to refine the questions before the main data collection?
6. "Since the participants were young, informed consent was obtained from all participants in this study... We obtained verbal consent." Given the involvement of minors in the study, could you explain why verbal consent was deemed sufficient? What additional measures were taken to ensure the ethical treatment of these participants, especially concerning their comprehension of the study's aims and their rights?
7. "The confirmatory factor analysis (CFA) was used to evaluate whether the observed variables accurately represent the constructs... Structural Equation Modeling (SEM) explored the structural relationships." Can you provide details on the model fit indices used in CFA and SEM, and what benchmarks were used to determine a good fit? How were the models adjusted based on the initial outcomes of these analyses?

**Results:**

1. The demographic distribution seems heavily skewed towards male participants (61.2%). Could this gender imbalance influence the generalizability of your results? How might this skew impact the interpretations of the effectiveness of various training technologies?
2. Your models display good fit indices (CFI, TLI, IFI, and RMSEA). However, RMSEA being slightly above the ideal threshold (0.057 vs. <0.05) suggests marginal discrepancies. Could you discuss potential model specifications or data characteristics that might have led to this outcome?
3. The validity and reliability of the constructs seem to be well within acceptable ranges, with Cronbach’s alpha and Composite Reliability all above 0.70, and AVEs mostly above 0.50. Could there be any specific factors or methodologies in your study design that particularly contributed to these strong reliability outcomes?
4. While most hypotheses about the technologies (AI, VR, PT) showed significant positive effects on technical skills acquisition, the hypothesis regarding the weekly training plan (H1) did not. Is there a theoretical or practical explanation you could provide that might explain the ineffectiveness of weekly training plans? The negative and insignificant impact of personalized learning (H6) is intriguing. Can you delve deeper into why you think personalized learning did not show the expected mediation effect? Are there limitations within the study or the implementation of personalized learning that could have influenced these results?
5. None of the mediation effects hypothesized were supported in the analysis. This is a critical finding. Could you discuss whether the constructs used for measuring personalized learning were adequately capturing the intended aspects of personalization? What modifications to the personalized learning implementation might better capture its potential mediating effects in future studies?
6. While many relationships were statistically significant, the practical significance (effect sizes) also matters in interpreting the results. For instance, although the effect sizes are reported in some cases, discussing their practical implications in real-world training scenarios would enhance the usefulness of the findings. How do these effect sizes translate into practical training changes?

**Discussion:**

1. The study highlights that physical training frequency significantly enhances technical skills acquisition. Could you elaborate on the specific aspects or types of physical training that were most beneficial? Are there certain intensities or frequencies that correlate more strongly with improved performance?
2. You mentioned that weekly training plans were found to have an insignificant influence on skills acquisition. Could you provide more insight into the potential reasons why these plans did not yield significant results? Were there variations in the design or implementation of these plans across the participant group?
3. Virtual reality training environments were noted to have the highest influence among the technologies studied. Can you discuss the features of VR training that specifically contribute to its effectiveness compared to AI-powered systems and wearable sensors?
4. AI-powered coaching systems seem to offer substantial benefits by providing real-time feedback and performance analysis. How do coaches and trainers perceive the integration of these systems into their regular training routines? Is there resistance or challenges that need to be addressed to optimize the use of AI in sports training?
5. Although wearable sensors had the least effect among the technologies, they still play a crucial role in monitoring and feedback. Could you explore why these devices had a lesser impact? Might there be a lag in the translation of data collection to actionable training insights that could be optimized?
6. The insignificant effect of personalized learning contrasts with other literature that supports its effectiveness. Could this discrepancy be due to the methodologies used to implement personalized learning in this study? Are there qualitative data or feedback from participants that could shed light on their experiences with personalized training methods?
7. The safety benefits of VR training environments are highlighted, but could you delve deeper into how these environments prevent wear and tear compared to traditional methods? What specific elements of VR training reduce physical strain?

**Conclusion**:

1. Directly link the conclusions to specific results discussed in the study to reinforce the evidence-based nature of the conclusions.
2. Explicitly mention the limitations of the study, discussing how they may affect the interpretation of the results and the generalizability of the conclusions.
