# Supplementary material for: Optimizing young tennis players’ development: Exploring the impact of emerging technologies on training effectiveness and technical skills acquisition
Source: PLoS One. 2024 Aug 7;19(8):e0307882. doi: 10.1371/journal.pone.0307882 (PMC11305591; doi:10.1371/journal.pone.0307882)
Supplement: Attachment — Submitted filename: Review Comments Response.docx [file pone.0307882.s003.docx]

**Response to** **Editor and Reviewer comments**

**EDITOR REVIEW COMMENTS**

**Authors response**

The authors thank the editor and the reviewers of our manuscript titled "Optimizing Young Tennis Players' Development: Exploring the Impact of Emerging Technologies on Training Effectiveness and Technical Skills Acquisition" for the great review. We will attempt to respond to the questions raised by the editor and the reviewers.

**Specific Comments:**

**Abstract**：Could you expand on the results in the abstract to include specific statistical details? This would provide a clearer and more comprehensive picture of how each technology influenced technical skills acquisition and the extent to which personalized learning did not have a significant mediation effect.

**Response:** The abstract has been rewritten to include specific statistical details including the model fitness results and the hypotheses testing.

**Introduction**：It is recommended to reorganize the introduction. Could expanding the discussion on existing research and literature more clearly demonstrate the specific knowledge gaps your study addresses? There is a need to more tightly connect the shortcomings of traditional methods with the advantages of new technologies. Please clearly indicate how each emerging technology overcomes specific limitations of traditional training, including detailed application examples, thereby enhancing the article's arguments.

**Response:** The section on “Challenges of traditional training methods” and “Emergence and potential of new technologies in tennis” discussed examples of the shortcomings of traditional training methods and how emerging technologies is filling the gap covering the limitations of traditional training methods and how they have been enhanced bt new technologies in tennis. Specific examples have been added of tennis players who have benefitted from emerging technologies.

1. "Physical conditioning is the other sporting attribute critical for tennis players. Many sports activities are physical, and players require proper strength and conditioning to ensure competition." What specific elements does 'physical conditioning' comprise? How does it impact the athletic performance of tennis players?

**Response:** the elements of physical conditioning have been added in in the introduction, including how it impacts athletes’ performance.

2. "Training through exercise and workouts among young players helps in developing speed, agility, strength, and coordination." Could you specify which types of 'exercise and workouts' are included here?

**Response:** paragraph has been included to include types of exercises and workouts that help tennis players develop speed, agility, strength and coordination.

3. "Traditional tennis sport training methods have long played a critical role in athlete development." Could you detail what encompasses traditional training methods? Please include necessary references.

**Response:** Traditional training methods include fundamental techniques such as serves, volleys, groundstrokes, and footwork in regular practice sessions that emphasize repetition and precision to build muscle memory and consistency. These have been outlined including supporting references (Gomez-Pinilla & HillmanN, 2013; Nugroho et al., 2023; Ericsson & Harwell, 2019; Zhang, 2024; Mason et al., 2020; Otte et al., 2020).

4. "However, emerging issues in sports training activities depict challenges inherent in traditional training methods." Could you provide a detailed explanation of "emerging issues," offering specific examples or data that support these challenges in traditional training methods?

**Response:** The emеrging issues have been further explained with specific examples relevant to the discussion like the Martina Hingis example and the report by Williams and Wigmore (2020) highlighting challenges athletes’ face during games including the pressure from fans and the game itself.

5. "Traditional training methods need help in replicating the real game scenarios, which can lead to differences in players' performance." You noted challenges with replicating real game scenarios using traditional training methods. Could you elaborate on how these limitations specifically affect the skill development of young tennis players?

**Response:** The example used was the Martina Hingis meltdown in the final of the 1999 French open where she lost after challenging the umpire and getting on the wrong side of the crowd. She never won another grand slam in her career. This is an example of where the pressure affected the growth of a 19 year old tennis prodigy.

6. "High physical activities during training increases the risk." Could you describe what types of injury risks are associated with high-intensity physical activities?

**Response:** We have added examples of the injuries “…foot-loading patterns that develop due to repetitive movements during intense tennis training; tennis players are also prone to shoulder injuries such as rotator cuff tears, impact, glenohumeral instability, and tendinopathy in the wrist due to the high amount of spin and speed they generate in their strokes.”

7. "Also, traditional training practices have limited rehabilitation integration." "Limited player rehabilitation integration can lead to slowed recovery from injury and return to top performance." How does traditional training impact rehabilitation integration? Is there any data or research to support this?

**Response:** Traditional recovery procedures often involve generalized procedures for anyone with suh an injury compared to customized dynamic procedures that are created specific for the athlete. A lot of data is considered before recommending a recovery strategy that is optimized for the athlete as Ayala et al. (2016) and Yildiz et al. (2019) identified.

8. "Some essential technologies include an AI-powered coaching system, which utilizes artificial intelligence to help coaches analyze and track players' performance." Could you provide specific examples of how AI systems analyze and track performance, and how this data is subsequently utilized to develop training plans?

**Response: “**Some essential technologies include an AI-powered coaching system, which utilizes artificial intelligence to help coaches analyze and track players' performance (Liu, 2021). Through AI-based technologies, coaches can gain insights into the players' strengths and weaknesses and consequently make necessary changes in training through personalized training.” This already answers your question. The study by Liu (2021) provides data that show how an AI is used by coaches to analyze player strengths and weaknesses and know where to optimize training to improve performance.

9. "Virtual Reality (VR) training environment is the other emerging technology in sports training that offers a dynamic and distraction-free environment." Can you provide examples of how VR technology creates a "distraction-free" environment and how this specifically benefits technical skill training?

**Response:** VR environments are simulations, thus, athletes can see themselves in action and assess their own strengths and weaknesses, and also try out new methods to see how it would turn out without risk to themselves in any way. VR environment are “distraction free” because only the athlete or those connected to the system will have access to the data the athlete is examining or watching the athlete perform in the virtual world where they can practice without external limitations.

10. "Wearable Sensor Technology in training offers players improved performance due to the ability to track health and fitness data, which is then used to enhance the individuals' athletic performance." How does Wearable Sensor Technology enhance athletic performance through tracking health and fitness data? Please provide a detailed explanation.

**Response:** Wearable devices monitor health and fitness data. This includes sleep times, steps taken in a day, sprint race times, heart rate, blood pressure etc. These devices work round the clock monitoring player performance during training and in real-game scenarios, which can then be analyzed to ensure that training performances are replicated during match plays. Any deviation is then investigated to understand the reason. For example, the coach can work with the player to ensure that they maintain the same blood pressure during training and game times, any deviations will lead to studying the data to understand why the blood pressure rises during games for example while lower during training situations. The coaches will then ensure to get the player in the same condition at all times to replicate their performance in training during match plays.

**Methods**

1. "From AI-powered coaching systems to virtual reality training environments and wearable sensors, training technologies play critical roles in skill refinement..."Could you provide more specific examples of how each technology—AI systems, VR environments, and wearable sensors—concretely contributes to skill refinement in tennis training? Are there particular skills these technologies target more effectively.

**Response:** Specific examples have been provided showing how AI systems, VR and wearable sensors impact players and the particular skills these technologies improve. References were also added to support them.

2. "The diffusion of Innovation Theory explains the phenomenon of emerging technologies in tennis training as an idea that gains traction and spreads to society." How does the Diffusion of Innovation Theory specifically apply to the adoption rates of these technologies among tennis coaches and athletes? Can you elaborate on how the characteristics of early adopters versus laggards manifest in this particular sports training context?

**Response:** The Diffusion of Innovation Theory, applied to adopting emerging technologies in tennis training, illustrates how tennis coaches and athletes progressively embrace AI-powered coaching systems, VR environments, and wearable sensors. Early adopters, typically innovative and risk-taking coaches, and athletes, quickly integrate these technologies to gain a competitive edge, utilizing AI for personalized training plans, VR for immersive practice sessions, and wearable sensors for detailed performance analytics. They value the potential for enhanced training efficiency and improved performance. In contrast, laggards are more traditional, skeptical of new technologies, and slower to change their established training routines, mainly due to the inability to meet the cost of the new technologies. They may rely on proven, conventional methods and adopt new technologies only when they become mainstream and demonstrate clear, widespread benefits. This disparity results in a gradual shift in training practices, with early adopters leading the way and demonstrating the advantages, eventually convincing laggards to follow suit as the technologies prove their efficacy in improving athletic performance. This can also be attributed to the fact that developing nations hardly have any top players in tennis; they are still relying on traditional methods, making it hard for their players to break into the top-tier of tennis.

3. "Various studies opine that those technologies significantly influence tennis athletes' training activities..."Are there contrasting studies that may not support the effectiveness of these technologies in enhancing tennis training? Including a range of studies might provide a balanced view and strengthen the argument for the adoption of these technologies.

**Response:** There are no studies disputing the impact of AI. It will be like going backwards to be against the use of AI, because in the real sense, AI is not really making the player better by giving them strength overnight, what it does is tell them what they are doing wrong and where they need to improve. For example, if the data for a 5-year period show that every morning a player takes tea without milk, he wins his tennis matches, AI will only point this data out and recommend that the player takes tea without milk on game days or sleeping patterns too. AI in this instance is more about player data and helping the player understand themselves better. Thus, more information will always lead to better outcomes.

4. "The research investigated optimizing young tennis players' development... The study targeted a representative sample of a maximum of 400 young tennis players." How was the sample size of 400 young tennis players determined to be sufficient for this study? What statistical power analysis was conducted to ensure this sample size could adequately detect the expected effects?

**Response:** The authors used the Krejcie and Morgan (10970) sample size table that recommends using 384 sample size for populations of one million and above. For uniformity, the authors increased the sample size to 400.

5. "Primary data was collected from the respondents using a structured questionnaire... The questions on latent variables were framed on a Likert scale format." How were the questionnaire items developed and validated to ensure they accurately measure the constructs of interest, such as training effectiveness and technical skill acquisition? Were any pilot tests conducted to refine the questions before the main data collection?

**Response:** The questionnaire items were developed already developed scales discussed in the empirical literature; the Weekly Training Plan were adapted from García-González (2014) and Xiao et al. (2023); questions on Physical Training Frequency were developed from Farrow and Reed (2010) and Deng (2023); AI Powered Coaching Systems questions were adapted from Bačić (2018) and Bodemer (2023); questions on VR Environments were modified from Bodemer (2023), Le Noury (2021) and Cossich et al. (2023); Wearable Sensors questions were revised from Seshadri et al. (2019), Taghavi (2019) and Liu and Zhang (2022); Personalized Learning questions were expounded from Koopmann et al. (2020), Larkin and Barkell (2022), Opstoel et al. (2019) and Koopmann (2020). Because the scales had been pretested in the previous studies, there was no need to pilot test the questionnaire.

6. "Since the participants were young, informed consent was obtained from all participants in this study... We obtained verbal consent." Given the involvement of minors in the study, could you explain why verbal consent was deemed sufficient? What additional measures were taken to ensure the ethical treatment of these participants, especially concerning their comprehension of the study's aims and their rights?

**Response:** Verbal consent was required to ensure the participants were comfortable answering the questions. The verbal consent was in addition to the ethics review board approval received from the study, and also explaining to the center managers and coaches the aim of the study.

7. "The confirmatory factor analysis (CFA) was used to evaluate whether the observed variables accurately represent the constructs... Structural Equation Modeling (SEM) explored the structural relationships." Can you provide details on the model fit indices used in CFA and SEM, and what benchmarks were used to determine a good fit? How the models were adjusted based on the initial outcomes of these analyses?

**Response:** The details of the model fit indices were provided in “Model Fitness Evaluation,” where the benchmarks for each index were identified and the supporting references for each benchmark included. There was no need for adjustments as the models were all good fits satisfying the threshold of being above 0.90.

**RESULTS**

1. The demographic distribution seems heavily skewed towards male participants (61.2%). Could this gender imbalance influence the generalizability of your results? How might this skew impact the interpretations of the effectiveness of various training technologies?

**Response:** The gender imbalance, with 61.2% male participants, could influence the generalizability of the results. This skew could affect the interpretations of the effectiveness of various training technologies in several ways:

***Training Response Variability***: Men and women may respond differently to training stimuli due to physiological and biomechanical differences. For instance, hormonal differences can impact muscle recovery and adaptation, potentially leading to different outcomes in physical conditioning and skill acquisition.

***Technology Interaction***: The interaction with training technologies like AI systems, VR environments, and wearable sensors may differ between genders. Previous studies have indicated that men and women can exhibit different levels of engagement and feedback preferences when using technology, which might affect the training outcomes observed.

***Generalizability of Findings***: Overrepresenting male participants’ means that the findings might not fully apply to female athletes.

Unfortunately, this is one of the limitations of this study. The suggestions for future studies will be to ensure that future studies take the gender representation of the population into account when administering the data collection instrument.

2. Your models display good fit indices (CFI, TLI, IFI, and RMSEA). However, RMSEA being slightly above the ideal threshold (0.057 vs. <0.05) suggests marginal discrepancies. Could you discuss potential model specifications or data characteristics that might have led to this outcome?

**Response:** 0.057 is still a good fit, however, the slight discrepancy may be attributed to the heterogeneity within the data, such as differing variances and covariances among subgroups (most likely the gender, age, or skill level), could affect the model fit. Any underlying patterns or anomalies not accounted for in the model specification might contribute to the higher RMSEA.

3. The validity and reliability of the constructs seem to be well within acceptable ranges, with Cronbach’s alpha and Composite Reliability all above 0.70, and AVEs mostly above 0.50. Could there be any specific factors or methodologies in your study design that particularly contributed to these strong reliability outcomes?

**Response:** The factors responsible for the strong reliability outcomes are mainly because the measurement instruments were developed thoroughly, including a comprehensive literature review and expert consultations to ensure that items accurately reflected the measured constructs. This careful design helped develop items consistently measuring the intended constructs, leading to higher reliability. We also ensured that each item in the survey was clear, specific, and easy to understand. Well-defined items related to the constructs help participants respond more accurately, increasing internal consistency.

4. While most hypotheses about the technologies (AI, VR, PT) showed significant positive effects on technical skills acquisition, the hypothesis regarding the weekly training plan (H1) did not. Is there a theoretical or practical explanation you could provide that might explain the ineffectiveness of weekly training plans? The negative and insignificant impact of personalized learning (H6) is intriguing. Can you delve deeper into why you think personalized learning did not show the expected mediation effect? Are there limitations within the study or the implementation of personalized learning that could have influenced these results?

**Response:** Their nature can explain the ineffectiveness of the weekly training plan (H1); it may lack the flexibility needed to adapt to athletes' evolving needs and performance levels. Tennis players, particularly young ones, often require dynamic and responsive training schedules that can be adjusted based on their immediate progress, fatigue levels, and skill acquisition rates. A rigid weekly plan might not accommodate these aspects. Weekly training plans are more about quantity than quality, repeating the same routines regardless of their effectiveness. Effective skill acquisition in tennis depends on the quality of training, including the precision of drills, the relevance of exercises to actual match play, and the feedback provided during training sessions. Without a focus on these qualitative aspects, a weekly training plan alone might not lead to significant improvements. Also, athletes have varied learning styles, physical capabilities, and developmental rates. A standardized weekly plan may not effectively cater to these individual differences, leading to suboptimal outcomes for many players.

As for the effects of the effectiveness of personalized learning, it hinges on how well it is implemented. If the personalized learning strategies were not adequately tailored to the athletes' specific needs and current skill levels, or if the implementation lacked consistency and depth, the expected benefits might not materialize. Personalized learning requires detailed and ongoing assessments to tailor the training accurately, which might have been a challenge in this study. Personalized learning plans can sometimes be challenging for coaches and players. The potential positive effects could be diluted if the athletes or coaches found it challenging to fully engage with the personalized plans due to their complexity or insufficient resources (e.g., time and technological tools). Also, personalized learning needs to be well-integrated with other training components to be effective.

These discussions have been well implemented in the study, there were no observed limitations due to personalized learning observed from the findings of the study.

5. None of the mediation effects hypothesized were supported in the analysis. This is a critical finding. Could you discuss whether the constructs used for measuring personalized learning were adequately capturing the intended aspects of personalization? What modifications to the personalized learning implementation might better capture its potential mediating effects in future studies?

**Response:** The results show that the mediation effects were not supported, indicating that implementing personalized learning requires adjustments to capture its potential mediating effects better. There may be a need for more robust algorithms and technologies that provide highly personalized training programs to be integrated based on a detailed analysis of players' performance, learning styles, and progress, which includes AI-driven adaptive learning systems that modify training content and difficulty in real-time. Another method of improving the effects of mediation is integrating personalized learning with other AI, VR, and wearable sensors for better individual tracking, health, and performance data. Training sessions should be customized in content and structure, allowing flexibility in timing, intensity, and type of exercises based on the player's evolving needs and responses. Future studies can mitigate this by using a mixed-method approach incorporating qualitative and quantitative data or using control groups that do not receive personalized learning interventions to isolate and measure the effects of personalization more clearly and also by including control groups that do not receive personalized learning interventions to isolate and measure the effects of personalization more clearly.

6. While many relationships were statistically significant, the practical significance (effect sizes) also matters in interpreting the results. For instance, although the effect sizes are reported in some cases, discussing their practical implications in real-world training scenarios would enhance the usefulness of the findings. How do these effect sizes translate into practical training changes?

**Response:** The effect sizes brief explanations have been included in the result presentation. They are highlighted below:

**Physical Training Frequency (PT)**

The effect size for physical training frequency (β = 0.198, p=0.000) indicates a meaningful positive impact on technical skills acquisition. In practical terms, this suggests that increasing the frequency of physical training sessions can substantially enhance players' technical skills. Coaches should consider incorporating more frequent, varied physical exercises to boost overall performance. This could include a mix of strength training, endurance workouts, and flexibility exercises to cover all aspects of physical fitness.

**AI-Powered Coaching Systems (AI)**

The effect size for AI-Powered Coaching Systems (β = 0.349, p=0.000) is notably significant, highlighting the benefits of integrating AI into training regimens. In practical terms, AI systems can offer personalized feedback, analyze players' performances in real time, and suggest tailored drills and exercises. This can lead to more efficient skill acquisition and refinement. Coaches and training facilities should invest in AI technologies to provide players with advanced, data-driven insights and personalized coaching.

**Virtual Reality Training Environments (VR)**

The substantial effect size for Virtual Reality Training Environments (β = 0.476, p=0.000) indicates their highly influential role in skill development. VR can simulate match conditions and scenarios, allowing players to practice and hone their skills in a controlled, immersive environment. This technology can be particularly beneficial for mental conditioning and strategy planning, enabling players to visualize and practice their responses to various in-game situations without actual play's physical wear and tear.

**Wearable Sensors (WS)**

Wearable sensors also show a significant effect size (β = 0.171, p=0.000), underscoring their utility in monitoring and enhancing performance. These devices can track various physiological and biomechanical metrics, providing real-time feedback on movement efficiency, injury risk, and overall physical condition. Coaches can use this data to adjust training loads, prevent injuries, and optimize performance. For players, wearable sensors can offer insights into their training progress and areas needing improvement.

**Discussion**

1. The study highlights that physical training frequency significantly enhances technical skills acquisition. Could you elaborate on the specific aspects or types of physical training that were most beneficial? Are there certain intensities or frequencies that correlate more strongly with improved performance?

**Response:** More explanation has been provided on the different types of physical training and the frequency that correlate to the frequency that leads to improved performance, they include “high-intensity interval training (HIIT), strength training, agility drills, and endurance exercises were particularly beneficial.”

2. You mentioned that weekly training plans were found to have an insignificant influence on skills acquisition. Could you provide more insight into the potential reasons why these plans did not yield significant results? Were there variations in the design or implementation of these plans across the participant group?

**Response: “**Several aspects could be pointed out as to why the weekly training plan was insignificant in technical skills acquisition. First, it suggests that developing a training plan without proper consideration of the quality of training would not bear desirable results as well as the flexibility needed to adapt to athletes’ evolving needs and performance level. It emphasizes that the quality of training is more crucial than the quantity of training sessions. Tennis players, particularly young ones, often require dynamic and responsive training schedules that can be adjusted based on their immediate progress, fatigue levels, and skill acquisition rates. A rigid weekly plan might not accommodate these aspects.” This has been used in the discussion to explain the insignificant influence of weekly plans on skills acquisition.

3. Virtual reality training environments were noted to have the highest influence among the technologies studied. Can you discuss the features of VR training that specifically contribute to its effectiveness compared to AI-powered systems and wearable sensors?

**Response:** “VR training environments excel due to their immersive and interactive nature, allowing players to simulate real match conditions with high fidelity. This enables focused practice on specific skills in a controlled, distraction-free setting. “ This has been expanded in the study to address the questions you raised.

4. AI-powered coaching systems seem to offer substantial benefits by providing real-time feedback and performance analysis. How do coaches and trainers perceive the integration of these systems into their regular training routines? Is there resistance or challenges that need to be addressed to optimize the use of AI in sports training?

**Response:** More context has been provided in the discussion section. This is a snippet of the added discussion “Coaches and trainers generally perceive AI-powered coaching systems as valuable tools for enhancing training routines by offering real-time feedback and detailed performance analysis; these systems help in identifying strengths and weaknesses, enabling personalized and data-driven training programs (Bridgeman & Giraldez-Hayes, 2024; Glebova et al., 2024).”

5. Although wearable sensors had the least effect among the technologies, they still play a crucial role in monitoring and feedback. Could you explore why these devices had a lesser impact? Might there be a lag in the translation of data collection to actionable training insights that could be optimized?

**Response:** Several factors were proposed for the reason of their lesser impact, one is the potential lag in translating collected data into actionable insight, the volume of the collected data, and the time required to analyze the data all combine to make it less attractive for coaches. This has been well explained in the discussion.

6. The insignificant effect of personalized learning contrasts with other literature that supports its effectiveness. Could this discrepancy be due to the methodologies used to implement personalized learning in this study? Are there qualitative data or feedback from participants that could shed light on their experiences with personalized training methods?

**Response:** More explanation has been provided in the discussion, this has also been identified as one of the study limitations as there is need to include qualitative studies in the future to get perspectives from athletes and coaches about deploying personalized learning in improving athlete performance.

7. The safety benefits of VR training environments are highlighted, but could you delve deeper into how these environments prevent wear and tear compared to traditional methods? What specific elements of VR training reduce physical strain?

**Response:** This has been adequately addressed in the literature and discussion. VR training prevents wear and tear as the athlete could use a very comfortable setting, like their bedroom or a designated space to simulate real-life scenarios without actually being physical. For example, the crowd noise can be increased to gauge player reaction, weather conditions can also be improvised like wind and rain to see player reaction to these scenarios without physically standing in the rain or in the open where win is blowing. It give the player an idea of his performance under these conditions and the coaches can use the data to better train their players. The fact that the player is not physically exerting himself during the process helps to reduce the physical strain on them. The min strain they face during VR training is the mental strain.

**Conclusion**:

1. Directly link the conclusions to specific results discussed in the study to reinforce the evidence-based nature of the conclusions.

**Response:** The conclusions have been directly linked to the study findings as suggested. Thank you for this insight, we appreciate.

2. Explicitly mention the limitations of the study, discussing how they may affect the interpretation of the results and the generalizability of the conclusions.

**Response:** The study limitations have been thoroughly discussed and included in the conclusion section. Thank you.

**Additional Editorial Requirements**

When submitting your revision, we need you to address these additional requirements.

1. Please ensure that your manuscript meets PLOS ONE's style requirements, including those for file naming. The PLOS ONE style templates can be found at https://journals.plos.org/plosone/s/file?id=wjVg/PLOSOne_formatting_sample_main_body.pdf and https://journals.plos.org/plosone/s/file?id=ba62/PLOSOne_formatting_sample_title_authors_affiliations.pdf

**Response:** The manuscript has been prepared to meet the editorial requirements of PloS ONE journal.

2. Please note that PLOS ONE has specific guidelines on code sharing for submissions in which author-generated code underpins the findings in the manuscript. In these cases, all author-generated code must be made available without restrictions upon publication of the work. Please review our guidelines at https://journals.plos.org/plosone/s/materials-and-software-sharing#loc-sharing-code and ensure that your code is shared in a way that follows best practice and facilitates reproducibility and reuse.

**Response:** The code sharing guidelines have been followed as specified.

3. In the ethics statement in the Methods, you have specified that verbal consent was obtained. Please provide additional details regarding how this consent was documented and witnessed, and state whether this was approved by the IRB

**Response: Additional update has been provided on the IRB approval. The detailes have been included in the methods section.**

4. Thank you for stating the following in your Competing Interests section: "No authors have competing interests"

Please complete your Competing Interests on the online submission form to state any Competing Interests. If you have no competing interests, please state "The authors have declared that no competing interests exist.", as detailed online in our guide for authors at http://journals.plos.org/plosone/s/submit-now This information should be included in your cover letter; we will change the online submission form on your behalf.

**Response: This will be done at the point of resubmission. Thank you.**

5. PLOS requires an ORCID iD for the corresponding author in Editorial Manager on papers submitted after December 6th, 2016. Please ensure that you have an ORCID iD and that it is validated in Editorial Manager. To do this, go to ‘Update my Information’ (in the upper left-hand corner of the main menu), and click on the Fetch/Validate link next to the ORCID field. This will take you to the ORCID site and allow you to create a new iD or authenticate a pre-existing iD in Editorial Manager. Please see the following video for instructions on linking an ORCID iD to your Editorial Manager account: <https://www.youtube.com/watch?v=_xcclfuvtxQ>

**Response: T**he Orcid numbers for the authors have been included in the author information.

**REVIEWERS COMMENTS**

**Reviewer #1:**

Thank you for the opportunity to review your manuscript titled "Optimizing Young Tennis Players' Development: Exploring the Impact of Emerging Technologies on Training Effectiveness and Technical Skills Acquisition." This study addresses an important area in sports science, focusing on how emerging technologies can be integrated into the training programs of young athletes to enhance their performance.

**Response:** Thank you for your kind words.

**General Comments:**

Your manuscript is well-structured and introduces an innovative angle on sports training technologies. However, the manuscript would benefit significantly from a deeper analysis and clearer exposition of some key aspects:

**Response:** We will follow all your recommendations.

**Statistical Analysis and Results Interpretation:**

The results section provides a broad overview of findings but lacks depth in the interpretation of these results. It would be beneficial to include a more detailed discussion on how these findings compare with existing literature, especially concerning the effectiveness of different technologies.

**Response:** A more rigorous interpretation and discussion of the statistical analysis has been included in the results section.

**Methodological Rigor:**

More detailed information on the methodological design would enhance the credibility of the study. Specifics regarding the participant selection process, the configuration of technologies used, and the statistical methods applied should be clearly articulated.

**Response**: A more detailed explanation of the methodological design has been presented to clarify and enhance the credibility of the research design.

**Ethical Considerations:**

Given that your study involves young participants, a more thorough explanation of the ethical considerations and consent procedures would be appropriate. Please clarify how informed consent was obtained from participants or their guardians and discuss any ethical approvals obtained for the study.

**Response:** The ethical considerations have been explained further and the IRB approval has been added to bolster the credibility of the data collection process. Thank you for reminding me this.

**Limitations and Future Research:**

While some limitations are briefly mentioned, a more comprehensive discussion would provide a better context for the findings. Additionally, suggestions for future research could be more specifically tied to the limitations and findings of the current study.

**Response**: More study limitations have been included and they have been tied to future studies.

**Research and Publication Ethics:**

No concerns about dual publication or research ethics have been identified based on the review. However, ensuring that all data presented is original and not previously published is crucial. If any part of the study overlaps with previous publications, those should be cited appropriately.

**Response**: No part fot he study overlaps with previous publications. The data collected were specifically for the study.

**Overall Recommendation:**

I recommend that the manuscript be considered for publication after major revisions. The suggested changes will strengthen the argument, improve the readability, and enhance the manuscript’s contribution to the field of sports science and athletic training. These comments are intended to guide you in strengthening your manuscript and do

**Response**: The suggested changes have been included as suggested. We appreciate the time taken to read our work and constructively offer feedback.

These comments are intended to guide you in strengthening your manuscript and do not detract from the importance and timeliness of your research. I look forward to seeing the revised version of your work.

**Response:** Thank you for your review.

**Reviewer #2: Review comment**

This manuscript entitled “Optimizing Young Tennis Players' Development: Exploring the Impact of Emerging Technologies on Training Effectiveness and Technical Skills Acquisition” primarily aimed to evaluate the impact of emerging technologies on the effectiveness of training and technical skills acquisition among young tennis players. The results of this study provide guidance for human movement science and sports science. While it is a very interesting topic. But I think this manuscript has some flaws to fill in before it can be published in a journal. There are several questions should be addressed, which list below. I give a major revision for this manuscript. Specific comments

1. In the Abstract part, " The rapid advancement in sports science and technology has replaced traditional training methodologies..." The statement implies a complete replacement of traditional methods. Can the authors clarify if any traditional training methodologies are still effective or relevant in current sports science, particularly in tennis, despite the advancements mentioned.

**Response**: The part about traditional training methodologies being replaced has been deleted. Traditional methods have not been ideally replaced, rather, they have been integrated with with new technologies to enhance training efficiency. Technology helps identify weaknesses and athletes can better work to improve on those areas instead of just training to improve general skills and performance which is what happens with traditional training methods.

2. In the introduction part, “According to Kolman , sports activities, including tennis, require skills to ensure high performance.” The introduction discusses skills required for high performance in tennis. Could the authors expand on how these skills are categorized (e.g., psychological, physiological, technical) and their relative impact on performance.

**Response:** Skills in tennis have been categorized as suggested and their impact on performance has been expounded.

3. “Training through exercise and workouts among young players helps in developing speed, agility, strength, and coordination.” Please use more then one references to support this sentence.

**Response:** Two more citations have been included to support the view (Huang et al., 2023; Nygaard Falch & Guldteig Rædergård, 2019)

4. “Telemetry sensors involve devices that keep track of the players' technique through keeping track of real-time data analysis” Symmetry training is also an important ability in tennis, and the reviewers suggest that the authors add relevant references. (DOI: 10.5334/paah.215).

**Response:** The reference has been included as suggested. Thank you.

5. In the Materials and methods part, Given the quantitative approach, how do the authors ensure that the subjective nature of self-reported data does not bias the outcomes, particularly in assessing the effectiveness of AI and VR technologies in training.

**Response:**

In the Materials and Methods section, the authors mitigate the potential bias from self-reported data by incorporating several strategies to ensure the objectivity and reliability of the outcomes in assessing the effectiveness of AI and VR technologies in training:

1. The authors use validated and standardized questionnaires previously tested for reliability and accuracy in similar contexts, ensuring that the responses are consistent and comparable.
2. The authors use blinding techniques, in which participants are unaware of the specific hypotheses being tested, minimizing response bias influenced by participants' expectations.
3. They conduct statistical analyses to identify and control for potential confounding variables, ensuring that the observed effects are attributable to the AI and VR interventions rather than extraneous factors.

These methodological safeguards collectively enhance the validity and reliability of the study's findings.

6. In the discussion part. "Virtual reality training environments were found to have the highest influence..." The manuscript highlights the significant impact of VR environments. Could the authors speculate on the potential long-term effects of VR training on athletes' performance, particularly regarding skill transfer to real-world settings.

**Response:** The discussion on VR environments and its impact on athlete performance has been further discussed in the results and discussion sections. Thank you for pointing this out.

7. In the Conclusion part, "The research indicated that emerging technologies are vital..." In concluding that emerging technologies are vital, how do the authors address the challenges or limitations that these technologies present in practical training settings.

**Response**: The conclusion section has been expanded to include study limitations and implications for future study.
