# Supplementary material for: Optimizing young tennis players’ development: Exploring the impact of emerging technologies on training effectiveness and technical skills acquisition
Source: PLoS One. 2024 Aug 7;19(8):e0307882. doi: 10.1371/journal.pone.0307882 (PMC11305591; doi:10.1371/journal.pone.0307882)
Supplement: Attachment — Submitted filename: PLOSONEpeer-review.docx [file pone.0307882.s004.docx]

**Review comment**

Thank you for addressing the comments and making the necessary revisions to your manuscript titled "Optimizing Young Tennis Players' Development: Exploring the Impact of Emerging Technologies on Training Effectiveness and Technical Skills Acquisitions." I have carefully reviewed the revised version of the manuscript and am pleased to inform you that I am satisfied with the revisions made. The manuscript has significantly improved in terms of clarity, content, and structure.

The manuscript now includes more precise statistical details, which enhance the clarity of the results related to the effectiveness of AI-powered coaching systems, virtual reality training environments, and wearable sensors in sports training. The added examples and empirical data significantly strengthen the argument by illustrating the practical applications and benefits of these technologies in enhancing athletic performance.

The authors have adeptly addressed all the concerns I raised in my initial review, specifically by elaborating on the methodologies used and providing a deeper discussion on the potential of new technologies to overcome the limitations of traditional training methods. The comprehensive revision not only improves the manuscript's academic rigor but also enhances its relevance to contemporary training practices.

Considering these enhancements and the manuscript's alignment with the editorial standards of [PLOS ONE], I am pleased to recommend its acceptance for publication. The revised document is not only methodologically sound but also rich in content, making a significant contribution to the literature on sports science and athlete development.

Once again, I commend your efforts in revising the manuscript and thank you for considering my comments. I recommend accepting the manuscript for publication in its current form.
